# Does Land Degradation Increase Poverty in Developing Countries?

**DOI:** 10.1371/journal.pone.0152973

**Published:** 2016-05-11

**Authors:** Edward B. Barbier, Jacob P. Hochard

**Affiliations:** 1 Department of Economics and Finance, University of Wyoming, Laramie, Wyoming, United States of America; 2 Department of Economics and Institute for Coastal Science and Policy, East Carolina University, Greenville, North Carolina, United States of America; University of Maryland at College Park, UNITED STATES

## Abstract

Land degradation is a global problem that particularly impacts the poor rural inhabitants of low and middle-income countries. We improve upon existing literature by estimating the extent of rural populations in 2000 and 2010 globally on degrading and improving agricultural land, taking into account the role of market access, and analyzing the resulting impacts on poverty. Using a variety of spatially referenced datasets, we estimate that 1.33 billion people worldwide in 2000 were located on degrading agricultural land (DAL), of which 1.26 billion were in developing countries. Almost all the world’s 200 million people on remote DAL were in developing countries, which is about 6% of their rural population. There were also 1.54 billion rural people on improving agricultural land (IAL), with 1.34 billion in developing countries. We find that a lower share of people in 2000 on DAL, or a higher share on IAL, lowers significantly how much overall economic growth reduces poverty from 2000 to 2012 across 83 developing countries. As the population on DAL and IAL in developing countries grew by 13% and 15% respectively from 2000 to 2010, these changing spatial distributions of rural populations could impact significantly future poverty in developing countries.

## Introduction

Land degradation is a major problem globally, and is increasingly linked to food insecurity [1–3], vulnerability to climate change [1, 4–6] and poverty [7–10]. Since the 1980s, remotely sensed normalized difference vegetation index (NDVI) and net primary productivity (NPP) data have been used to determine the spatial distribution of populations on degrading land [8, 10–12]. Estimates indicate that over 1.5 billion people are affected by land degradation [10], and could include large numbers of the rural poor [7–9]. Overall poverty in developing countries may therefore be influenced by the concentration of rural populations on degrading, as opposed to improving, agricultural land [7, 13–15].

This study adds to past efforts to link land degradation to poverty in two ways. First, we offer an improved global data set that enables tracking progress to combat degradation over time (2000 and 2010); covers both the shares of rural population on degrading versus improving agricultural land; and classifies degrading versus improving agricultural land according to market access. Second, we conduct an analysis of the links between poverty and the share of rural populations on degrading versus improving agricultural land.

We use a variety of spatially referenced datasets to estimate the total numbers of rural people globally in 2000 and 2010 located on degrading versus improving agricultural land. Following [10, 11], we denote agricultural land to be degrading if it displays a negative change in NPP from 1981–2000, whereas it is improving if there is a non-negative change in NPP from 1981–2000. Market accessibility was also used to identify the spatial distribution of rural populations on remote degrading or improving agricultural land, where we define market access as less than five hours of travel to a city with a population of 50,000 or more, according to [16].

In addition, we examine whether the various spatial distributions of rural populations on degrading and improving agricultural land in 2000 affect changes in the rate of poverty from 2000 to 2012 in 83 developing countries, which are all low and middle-income economies with 2012 per capita income of US$12,615 or less [17]. Following [18], we test whether this influence on poverty is direct, or whether it occurs through altering the poverty-reducing impact of income growth.

## Results

### Rural Population on Degrading and Improving Agricultural Land

In 2000, there were 1.33 billion people worldwide located on degrading agricultural land (DAL) (Table 1 and Figure A in S1 File). Only 73 million were in high-income countries, whereas 1.26 billion were found in developing countries, around 32% of the rural population. This share ranged from 13% in Latin America & the Caribbean to 51% in East Asia & Pacific. Almost all the world’s 200 million people on remote DAL were in developing countries, which is about 6% of their rural population. This share ranged from 2% in Latin America and the Caribbean to 9% in East Asia and Pacific.

**Table 1:** Distribution of global rural population on degrading agricultural land, 2000–2010.

	Population in 2000 (millions)					% change from 2000 to 2010		
	Rural population (1)	Rural population on all DAL (2)	% share (2)/(1)	Rural population on all remote DAL (3)	% share (3)/(1)	Rural population (4)	Rural population on all DAL (5)	Rural population on all remote DAL (6)
**Developing country**	**3,706.8**	**1,258.7**	**32.4%**	**202.2**	**5.5%**	**14.6%**	**13.3%**	**13.8%**
East Asia & Pacific	1,398.4	710.3	50.8%	125.2	9.0%	7.2%	8.4%	6.8%
Europe & C. Asia	173.8	67.0	38.5%	6.2	3.6%	4.0%	1.0%	4.4%
Latin America & Caribbean	294.1	38.3	13.0%	5.6	1.9%	14.3%	18.4%	17.1%
Middle East & N. Africa	195.6	43.7	22.3%	5.4	2.8%	21.3%	14.3%	5.9%
South Asia	1,090.4	285.2	26.2%	27.4	2.5%	17.8%	17.8%	18.9%
Sub-Saharan Africa	554.6	114.1	20.6%	32.4	5.8%	28.%	37.8%	39.3%
**Developed country**	**404.7**	**72.6**	**17.9%**	**3.2**	**0.8%**	**2.6%**	**-2.8%**	**-1.8%**
**World**	**4,111.5**	**1,331.3**	**34.0%**	**205.4**	**5.0%**	**13.4%**	**12.4%**	**13.6%**

Degrading agricultural land (DAL) consists of agricultural land with a negative change in Net Primary Productivity (NPP) from 1981–2000. NPP is measured as the change in grams of carbon sequestered per square meter over the 1981–2000 time period after subtracting respiration losses. Market accessibility is used to identify remote DAL, where market access is defined as less than five hours of travel to a market city with a population of 50,000 or more [13]. Developing countries are all low and middle-income economies with 2012 per capita income of US$12,615 or less [17]. Column (1) is estimated for 205 countries. Columns (2) and (3) are estimated for 183 countries; one country was indeterminate due to changing political boundaries, and 21 countries had missing data or insufficient spatial resolution denoting agricultural land. Full details of the spatially referenced datasets used and methods used to derive these estimates are provided in the Materials and Methods and the S1 File Supporting Information.

From 2000 to 2010, in developing economies, the numbers on all and remote DAL grew 13% and 14% respectively, keeping pace with the overall growth in rural population (Table 1). However, these growth rates vary significantly by region, with the lowest increase occurring in Europe & Central Asia (1–4%) and the highest in Sub-Saharan Africa (17–18%). In contrast, in high-income countries, the rural population on all DAL fell by 3%, and declined by 2% on remote DAL. By 2010, there were 1.5 billion people on DAL globally, and 1.4 billion in developing countries (Table A in S1 File and Figure B in S1 File). They comprised 32% of the rural population worldwide and nearly 34% in low and middle-income economies. The rural population on remote DAL in 2010 was over 230 million, and located almost entirely in developing countries. They accounted for around 5% of the rural population (Table A in S1 File and Figure B in S1 File).

In 2000, there were around 1.5 billion people on improving agricultural land (IAL), with 1.3 billion in developing countries (Table 2 and Figure C in S1 File). People on IAL constituted 37% of the rural population worldwide and 36% in developing economies. Just over 160 million people on IAL were without market access, almost all in developing countries. They accounted for about 4% of rural populations worldwide.

**Table 2:** Distribution of global rural population on improving agricultural land, 2000–2010.

	Population in 2000 (millions)					% change from 2000 to 2010		
	Rural population (1)	Rural population on all IAL (2)	% share (2)/(1)	Rural population on all remote IAL (3)	% share (3)/(1)	Rural population (4)	Rural population on all IAL (5)	Rural population on all remote IAL (6)
**Developing country**	**3,706.8**	**1,340.7**	**36.2%**	**155.3**	**4.2%**	**14.6%**	**14.8%**	**8.9%**
East Asia & Pacific	1,398.4	398.7	28.5%	67.9	4.9%	7.2%	11.9%	0.4%
Europe & C. Asia	173.8	66.7	38.4%	6.6	3.8%	4.0%	-0.6%	6.4%
Latin America & Caribbean	294.1	90.6	30.8%	9.3	3.2%	14.3%	14.1%	12.6%
Middle East & N. Africa	195.6	28.1	14.4%	1.7	0.9%	21.3%	23.0%	49.1%
South Asia	1,090.4	641.8	58.9%	37.3	3.4%	17.8%	14.4%	17.3%
Sub-Saharan Africa	554.6	114.8	20.7%	32.5	5.9%	28.%	34.5%	14.6%
**Developed country**	**404.7**	**196.4**	**48.5%**	**9.0**	**2.2%**	**2.6%**	**-3.0%**	**0.1%**
**World**	**4,111.5**	**1,537.1**	**37.4%**	**164.3**	**4.0%**	**13.4%**	**12.5%**	**8.5%**

Improving agricultural land (IAL) consists of agricultural land with a non-negative change in Net Primary Productivity (NPP) from 1981–2000. NPP is measured as the change in grams of carbon sequestered per square meter over the 1981–2000 time period after subtracting respiration losses. Market accessibility is used to identify remote IAL, where market access is defined as less than five hours of travel to a market city with a population of 50,000 or more [13]. Developing countries are all low and middle-income economies with 2012 per capita income of US$12,615 or less [17]. Column (1) is estimated for 205 countries. Columns (2) and (3) are estimated for 183 countries; one country was indeterminate due to changing political boundaries, and 21 countries had missing data or insufficient spatial resolution denoting agricultural land. Full details of the spatially referenced datasets used and methods used to derive these estimates are provided in the Materials and Methods and in S1 File Supporting Information.

From 2000 to 2010, the rural population on all IAL increased by around 13%, and on remote IAL by nearly 9% (Table 2). However, in developing economies, the rural population on all IAL grew by 15%, with the fastest growth occurring in Sub-Saharan Africa (35%) and a slight decline of almost 1% in Europe and Central Asia. The population on remote IAL in developing countries increased at a slower pace, around 9%. The fastest growth (49%) occurred in the Middle East & North Africa. In East Asia & Pacific the population was largely unchanged. In high-income countries, the rural population on all IAL fell by 3%, and on remote IAL the population was almost unchanged. By 2010, there were 1.7 billion people worldwide on IAL, of which approximately 1.5 billion were in developing countries (Table B in S1 File and Figure D in S1 File). The number of people on IAL without market access increased to nearly 180 million in 2010, with 170 million in developing countries. The global and regional shares of the rural population on all and remote IAL did not change significantly from 2000.

In sum, the distribution of rural populations on DAL is overwhelmingly a developing country problem. The number of people in these locations has increased significantly from 2000 to 2010, both worldwide and in each major developing country region (Table 1). However, an encouraging trend is the growth in the population of developing countries on all IAL, even in some remote areas and in poor regions such as Sub-Saharan Africa. But there has also been growth in the rural population of developing countries on remote DAL from 2000 to 2010. This critical population group appears to be expanding by over 1% annually across the developing world, 2% annually in Latin America & Caribbean and South Asia, and 4% in Sub-Saharan Africa (Table 1).

**Fig 1:**
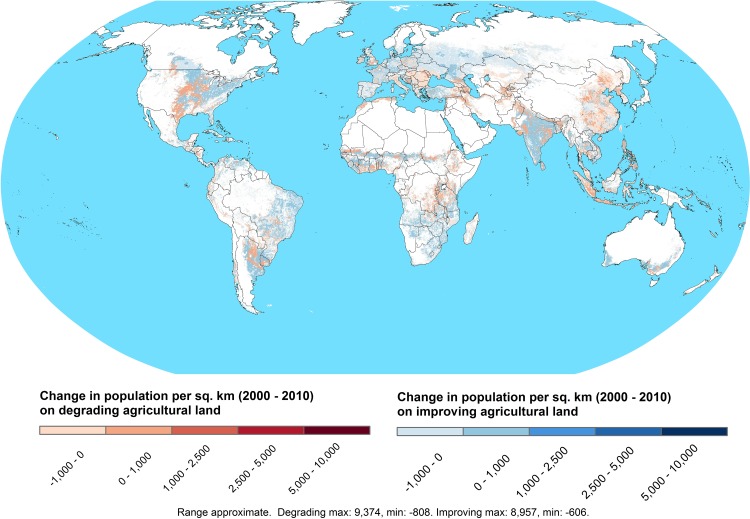
Change in population per km^2^ on degrading and improving agricultural land, 2000–2010. Degrading agricultural land (DAL) consists of agricultural land with a negative change in Net Primary Productivity (NPP) from 1981–2000. Climate-adjusted NPP is measured as the change in grams of carbon sequestered per square meter over the 1981–2000 time period after subtracting respiration losses. Improving agricultural land (IAL) consists of agricultural land with a non-negative change in Net Primary Productivity (NPP) from 1981–2000. NPP is measured as the change in grams of carbon sequestered per square meter over the 1981–2000 time period after subtracting respiration losses. Full details of the spatially referenced datasets used and the methods used to derive this figure are provided in the Material and Methods and the S1 File Supporting Information.

Fig 1 indicates the considerable change in the population density of rural populations on DAL and IAL over 2000 to 2010. Although the population density on IAL has increased significantly, so has the concentration of populations on DAL. All the major developing regions of the world indicate incidences of higher population densities on DAL since 2000, which suggests that this problem may be worsening (Fig 1).

### Impacts on poverty

Our analysis of whether the 2000 distribution of rural populations in 83 developing countries on DAL and IAL influences changes in poverty rates over 2000–2012 for these countries focuses on four spatial distribution variables:

- the share (%) of rural population located on all DAL (*d*_1_);
- the share (%) of rural population located on all remote DAL (*d*_2_);
- the share (%) of rural population located on all IAL (*i*_1_); and
- the share (%) of rural population located on all remote IAL (*i*_2_).

Table 3 summarizes the descriptive statistics for the key variables used in the poverty analysis for our sample of 83 countries (See also Table C in S1 File).

**Table 3:** Descriptive statistics of key poverty analysis variables.

	Descriptive Statistics		
Key variables	Mean	Median	Standard Deviation
Initial headcount poverty rate (% of population) in 2000, *H*	46.41	42.85	29.56
Annualized growth (%) in the poverty rate (US$2/day) from 2000–2012, γ(*H*)	-7.70	-4.26	10.28
Annualized growth (%) in the mean survey income per capita from 2000–2012, γ(μ)	3.36	3.32	3.52
Share (%) of rural population on all degrading agricultural land in 2000, *d*_1_	27.11	22.44	21.04
Share (%) of rural population on all remote degrading agricultural land in 2000, *d*_2_	5.02	3.81	4.43
Share (%) of rural population on all improving agricultural land in 2000, *i*_1_	31.89	29.6	21.05
Share (%) of rural population on all remote improving agricultural land in 2000, *i*_2_	13.45	5.21	18.83

Based on a sample of 83 developing countries. See Supporting Information.

For our sample of countries, we find no evidence that any of these four spatial distributions of the rural population in 2000 have a direct influence on poverty rates over 2000–2012. However, there is a statistically significant impact of these distributions through the poverty-reducing effects of income growth.

As previous poverty-growth analyses have shown [18–21], a rise in annual mean income growth reduces headcount poverty rates. But we find that this poverty-reducing effect is altered significantly when we adjust income growth over 2000 to 2012 for each of the initial spatial population distributions *d*_1_, *d*_2_, *i*_1_ and *i*_2_ (Table 3). A higher share of the rural population on DAL (*d*_1_ and *d*_2_) diminishes the poverty-reducing impact of per capita income growth, whereas a higher share of the rural population on IAL (*i*_1_ and *i*_2_) enhances this poverty-reducing impact of growth.

Consider an annual per capita income growth rate of 3.36%, which is the mean for the sample of 83 countries from 2000 to 2012 (Table 3). A country with a high share of rural population on degraded agricultural land *d*_1_, such as 48% (one-standard-deviation above the mean for all 83 countries), would expect to see a rate of poverty reduction of around 2.0 to 2.6% per year (Table 4). However, if 27% of a country’s rural population is on DAL (the mean for all 83 countries), then poverty would be reduced by 2.8% to 3.6% annually. Finally, if a country had only 6% of its rural population on degraded agricultural land (one-standard-deviation below the mean), then poverty would decline 3.7% to 4.6% each year. A similar pattern emerges for the share of rural agricultural population on remote DAL *d*_2_; as this share declines across countries, the poverty-reducing impact of income growth is enhanced.

**Table 4:** Effects of the distribution of rural population on degrading and improving agricultural land on the poverty-reducing impacts of growth in income per capita.

	Estimated parameters		Estimated parameters		Poverty-reducing impact of growth
Key spatial distribution variable	β_1_ (t-stat)	β_1_ (t-stat)	δ_1_ (t-stat)	δ_1_ (t-stat)	
Share (%) of rural population on all degrading agricultural land, *d*_1_	-2.15 (-3.83)	-2.51 (-4.39)	0.54 (8.91)	0.58 (7.05)	
*d*_1_ = 6.1%					-3.66% to -4.63%
*d*_1_ = 27.1%					-2.84% to -3.59%
*d*_1_ = 48.2%					-2.02% to -2.56%
Share (%) of rural population on all remote degrading agricultural land, *d*_2_	-2.31 (-4.72)	-2.91 (-6.42)	0.52 (12.62)	0.57 (10.90)	
*d*_2_ = 0.6%					-4.03% to -5.55%
*d*_2_ = 5.0%					-3.85% to -5.31%
*d*_2_ = 9.5%					-3.67% to -5.06%
Share (%) of rural population on all improving agricultural land, *i*_*1*_	-2.36 (-4.66)	-2.92 (-6.11)	0.34 (11.55)	0.37 (9.70)	
*i*_1_ = 10.8%					-2.99% to -4.02%
*i*_1_ = 31.9%					-3.33% to -4.48%
*i*_1_ = 52.9%					-3.60% to -4.85%
Share (%) of rural population on all remote improving agricultural land, *i*_2_	-2.30 (-4.34)	-2.86 (-5.60)	0.38 (10.11)	0.41 (8.47)	
*i*_2_ = 4.0%					-3.05% to -4.11%
*i*_2_ = 13.5%					-3.33% to -4.48%
*i*_2_ = 22.9%					-3.60% to -4.85%

The estimates of the poverty-reducing impact of growth in income per capita are *β*_1_(1 − *d*_*kj*_/100)*γ*(*μ*_*jt*_) for degrading agricultural land and *β*_1_(1 + *i*_*kj*_/100)*γ*(*μ*_*jt*_) for improving agricultural land, where the annualized growth rate in survey income per capita *γ*(*μ*) is evaluated at the mean for the sample of 83 countries, which is 3.36% (see Table 3). Parameter estimates for β_1_ and δ_1_ are from three-stage least squares (3SL3) estimations, with and without controls. *t*-ratios are in parentheses;**significant at the 1% level; *N* = 80. See Material and Methods, S1 File Supporting Information, and Tables D and E in S1 File for full details of the statistical analysis. The values for each spatial distribution variable correspond, respectively, to one-standard-deviation below the mean, the mean and one-standard-deviation above the mean for the sample of 83 developing countries in 2000 (see Table 3). The exception is the values for *i*_2_, which correspond, respectively, to one-half-standard-deviation below the mean, the mean and one-half-standard-deviation above the mean for the sample of 83 developing countries in 2000. All estimated parameters are significant at the 1% level.

In contrast, as a country’s share of the rural population on improving agricultural land increases, per capita income growth leads to more poverty reduction. With an annual per capita income growth rate of 3.36%, if the share of rural population on IAL is 11%, poverty declines by 3 to 4% annually (Table 4). In contrast, if the share rises to 32%, poverty will fall by 3.6% to 4.8% each year. Finally, if the share of rural population on IAL is 53%, poverty decreases by 4.1% to 5.6% per year. A similar effect occurs if a country’s share of rural population on remote IAL *i*_2_ rises.

## Discussion

Our results suggest that the concentration of rural populations on degrading agricultural land (DAL) is a major obstacle to the poverty-reducing effect of overall income growth in developing countries. However, if the share of the rural population on improving agricultural land (IAL) rises, then the poverty-reducing impact of growth is augmented.

These results support case study and cross-country evidence that rural populations living on degraded lands, especially in remote areas, may be insulated from the poverty-reducing effects of economy-wide growth [7, 13–15]. Persistent land degradation reduces the productivity of agricultural systems, on which many rural poor depend, thus trapping them in subsistence-level poverty. Geographical isolation raises substantially the costs of agricultural commerce and crop production in remote markets, distorts or insulates these markets from economy-wide policy changes, and thus discourages smallholder market participation and investment in improved farming systems and land management. Consequently, the income-generating benefits of economic growth may bypass poor households coping with land degradation, especially in remote locations with limited market access.

Thus, our findings suggest that there is a critical need to ensure that more rural people have improving, rather than degrading, agricultural land, especially in the remote regions of developing countries. This could be accomplished through a rural development strategy that invests in the enhancement of the livelihoods of agricultural households and market access in remote areas, improves agricultural land wherever possible, and if necessary, encourages out-migration of households on the DAL that is beyond improvement [7,13–15,22]. Such a strategy is an urgent priority, given that from 2000 to 2030 DAL is expected to increase worldwide by 1–2.9 million ha annually [5], and as we find, the rural population on DAL in developing countries has already increased by 13% from 2000 to 2010. Our finding that this growing problem is also preventing poverty reduction in developing countries should add to current concerns about the increasing vulnerability of the poor and their economic livelihoods to climate change [1,13,23].

To estimate improving and degrading agricultural land, we rely on standard spatially referenced datasets based on climate-adjusted NPP trends, i.e., the residual trend when controlling for changes in rainfall and temperature (see Materials and Methods and S1 File Supporting Information for more details). Although such NPP trends can reflect processes that can correspond to land degradation or improvement, remote sensing is necessarily limited to an evaluation of an aggregated outcome (vegetation cover) that is the result of various interacting biophysical and land use change factors on the ground [8]. Such an approach is also constrained by the availability of global data with sufficient detail and resolution. As further advances in spatial methods and global data improve, more accurate estimates of land degradation or improvement should result. Nevertheless, for the foreseeable future, for measuring changes in land degradation or improvement “as a proxy, the NDVI⁄NPP trend does provide a globally consistent yardstick, and it does highlight places where biologically significant change is happening” [10].

Our analysis of the impacts on poverty focuses on the share of the rural population on degrading or improving agricultural land. Other influences on poverty may arise from increasing population density or simply an absolute increase in the numbers of people on degrading versus improving agricultural land. Specific changes in land use cover, such as the conversion of forests to agricultural land, could cause significant changes in or accelerate processes of land degradation [5,7,9], as might the interplay of climatic factors and land degradation over time [1,2,6]. Finally, land degradation may have indirect impacts on poverty, through impacting agricultural productivity. Case studies in China, Ethiopia, Mexico, Uganda, Rwanda, Chile, and Indonesia estimated declines in overall agricultural productivity due to degradation of around 3–7% per year, an order of magnitude larger than the estimated cost of remediation [1].

## Materials and Methods

### Study Design

The objectives of this study are to estimate the extent of rural populations in 2000 and 2010 globally on degrading and improving agricultural land, including classifying these population distributions by market access, and then analyzing the resulting impacts on poverty. The latter statistical analysis examines whether the various spatial distributions of rural populations on degrading and improving agricultural land in 2000 affect changes in the rate of poverty from 2000 to 2012 in 83 developing countries

Our approach to the spatial analysis of rural populations on degrading and improving agricultural land over 1981–2000 follows the methods of previous studies [10,11], which depict global change using the normalized difference vegetation index (NDVI), scaled in terms of net primary productivity (NPP) change. Thus, in this analysis, *degrading agricultural land* consists of agricultural land with a negative change in net primary productivity from 1981–2000, where NPP is measured as the change in grams of carbon sequestered per square meter over the 1981–2000 time period after subtracting respiration losses. Consequently, *improving agricultural land* is agricultural land with a non-negative change in NPP from 1981–2000. Market accessibility was also used to identify *remote degrading* and *remote improving agricultural land*, where market access is less than five hours of travel to a market city with a population of 50,000 or more.

Using a variety of global spatially referenced datasets [24–28], we analyze the spatial distribution of rural populations across developing countries in 2000 and 2010 on degrading versus improving agricultural land over 1981–2000 (see S1 File Supporting Information for details of these data sets and sources). Degrading or improving land was determined using University of Maryland’s Global Land Cover Facility’s AVHRR Global Production Efficiency Model (GloPEM), which is available from 1981–2000 with annual summations of climate-adjusted net primary production (NPP) change measured in grams of carbon sequestered per square meter per year (gC/*m*^2^/yr) [24]. Agricultural land extent was obtained from the Pilot Analysis of Global Ecosystems (PAGE) [25], and rural populations determined from the rural-urban extent dataset that was published as part of CIESIN Global Rural Urban Mapping Project (GRUMPv1) [26]. Market accessibility was used to identify remote areas as released by the Global Environment Monitoring Unit of the Joint Research Centre of the European Commission, where market access is defined as less than five hours of travel to a market city with a population of 50,000 or more [16].

### Statistical Analysis

Our poverty analysis examines whether our four spatial distributions of rural populations in 2000 on degrading agricultural land, *d*_1_ and *d*_2_, and improving agricultural land, *i*_1_ and *i*_2_, affect changes in the rate of poverty from 2000 to 2012 in 83 developing countries. Following a standard approach in the literature [18], we test whether this influence on poverty is direct, or whether it occurs through influencing how income growth reduces poverty. We conduct both ordinary least squares (OLS) regressions, instrumental variables (IV), seemingly unrelated regressions (SUR) and three-stage least squares (3SLS). Full details of the estimation strategy and results of our poverty analysis can be found in the S1 File Supporting Information. Here, we summarize the key sources of data and steps for our approach.

We obtain our cross-country measures of a given poverty line *z*, the poverty headcount index *H*, and mean income μ from PovcalNet, the on-line tool for poverty measurement developed by the Development Research Group of the World Bank (Available online at http://iresearch.worldbank.org/PovcalNet/). PovcalNet produces internationally comparable country-level poverty and income distribution estimates based on more than 850 standardized household surveys across 127 developing countries. From this database, we identify 83 low and middle-income economies with at least two suitable household surveys from 2000 to 2012. The longest available spell between surveys is used for each country, and both surveys use the same welfare indicator, either consumption or income per person. The median interval between surveys is eight years, and it varies from two to eleven years. As far as possible, the initial survey year chosen was 2000, or for the soonest subsequent year. All monetary measures are in constant 2005 prices and are at Purchasing Power Parity (PPP).

The poverty headcount index *H* is the percentage of the population living in households with consumption per capita (or income when consumption is not available) below the poverty line. We follow a previous study [18] and choose a poverty line *z* of $2.00 per person per day at 2005 PPP, which is the median poverty line among developing countries. In the initial survey year, the median poverty headcount index across all 83 countries was 42.85%, but ranged widely from 0.29% to 95.44%. By the final survey year, the median poverty headcount was 27.86%, and it varied from 0.08% to 93.49%.

Mean income μ is the average monthly (2005 PPP $) per capita income or consumption expenditure from the household surveys for each country in the relevant year. In the initial survey year, the median per capita monthly income was $100 across all 83 countries, and ranged from $24 to $2,003. In the final survey year, median income was $115, and varied from $28 to $2,012.

We also employ a number of control variables in our analysis, following the approach of similar poverty analyses [18–21]. The controls are inflation, government consumption as a share of GDP, arable land per capita, agricultural value added as a share of GDP and per worker, investment as a share of GDP, trade openness, primary school enrollment, and life expectancy. These variables were obtained from the World Bank’s World Development Indicators (Available at http://databank.worldbank.org/data), and as far as possible, for 2000 and our sample of 83 countries. Other controls include a dummy for landlocked country as defined by UNDP (http://unctad.org/en/pages/aldc/Landlocked%20Developing%20Countries/List-of-land-locked-developing-countries.aspx), for small island developing states as defined by UNESCO (http://www.unesco.org/new/en/natural-sciences/priority-areas/sids/about-unesco-and-sids/sids-list/), and distance from equator for each country. We also employ rule of law and democracy (voice and accountability) indices, from the Worldwide Governance Indicators (http://data.worldbank.org/data-catalog/worldwide-governance-indicators), which were averaged over 1996–2000 for each country. Finally, we use regional dummies for the six main developing country regions, and the Gini index obtained from the PovcalNet surveys, as additional controls.To analyze the possible direct and indirect influences of our spatial distribution variables *d*_*k*_ and *i*_*k*_ in 2000 on poverty changes from 2000 to 2012 in our 83 sample countries, we follow a similar estimation strategy of a previous study [18]. The dependent variable of the regression is the annualized change in log headcount poverty rate for $2 a day between surveys, and thus represents growth in poverty. A standard hypothesis in poverty analysis is that changes in poverty over time will be influenced by growth in income [18–21]. That is, as the mean per capita income across surveyed households rises, one would expect their average poverty rate to fall. Thus, a key explanatory variable in determining changes in poverty between surveys is the annual growth in income per person, which is represented by the annualize change in log survey mean income between surveys. In this analysis, we are also interested in how each of our four spatial distribution variables in 2000 also influences the change in poverty over time. Each of these spatial distribution variables could have a direct impact on changes in poverty over time or it could affect the poverty-reducing impact of income growth. In all OLS, IV and 3SLS regressions, including those with and without various controls, tests on the restrictions of the regression coefficients confirm that our spatial distribution variables for DAL and IAL in 2000 do not have a direct influence on changes in poverty from 2000 to 2012 but they do influence indirectly changes in poverty from 2000 to 2012 through affecting the impact of income growth on poverty reduction.

## Supporting Information

S1 File Supporting Information. (DOCX)
