# Supplementary material for: Does Land Degradation Increase Poverty in Developing Countries?
Source: PLoS One. 2016 May 11;11(5):e0152973. doi: 10.1371/journal.pone.0152973 (PMC4864404; doi:10.1371/journal.pone.0152973)
Supplement: S1 File — (DOCX) [file pone.0152973.s001.docx]

**S1 File Supporting Information**

**Materials and Methods**

*Spatial analysis*

Our approach to the spatial analysis of rural populations on degrading and improving agricultural land over 1981-2000 follows closely that of previous studies [10, 11], which depict global change using the normalized difference vegetation index (NDVI), scaled in terms of net primary productivity (NPP) change. Thus, in this analysis, *degrading agricultural land* consists of agricultural land with a negative change in net primary productivity from 1981-2000, where NPP is measured as the change in grams of carbon sequestered per square meter over the 1981-2000 time period after subtracting respiration losses. Consequently, *improving agricultural land* is agricultural land with a non-negative change in NPP from 1981-2000. Market accessibility was also used to identify remote degrading and remote improving agricultural land, where market access is less than five hours of travel to a market city with a population of 50,000 or more [16].

*Data sources*

Several geospatial datasets were utilized in this analysis.

1. National boundaries were determined from the Gridded Population of the World, Version 3 (GPWv3): National Administrative Boundaries file as published by the Center for International Earth Science Information Network (CIESIN) and Centro Internacional de Agricultura Tropical (CIAT) in 2005 [26]. Country boundaries are denoted by polygons and are identified using unique ISO3V10 3-letter country/state codes. The geographic coordinates of this dataset are in decimal degrees using the World Geodetic System spheroid of 1984 (WGS84). Territories of countries were not included in this analysis.
2. Populations for 2000 and 2010 were identified using the Gridded Population of the World, Version 3 (GPWv3) dataset published in 2005 by the CIESIN, International Food Policy Research Institute (IFPRI) and CIAT [27]. It was decided not to use the higher resolution Global Rural-Urban Mapping Project (GRUMP), Version 1 also published by CIESIN because in addition to 1990, 1995 and 2000 population data, the GPWv3 also offers population projections for 2005, 2010 and 2015. The resolution of this GRID formatted raster is 0.041666667 by 0.041666667 decimal degrees or 2.5 by 2.5 arc-minutes (approximately 5 km^2^ cells).
3. Urban areas were identified using the Urban Extents Grid, Version 1 (1995) from GRUMP V1. This data was published in 2011 by CIESIN, IFPRI, the World Bank and CIAT [28]. The resolution of this GRID formatted raster is 0.0083333333 by 0.0083333333 decimal degrees or 30 arc-seconds (approximately 1 km^2^ cells). Rural areas were defined as those that are non-urban.
4. Global agricultural lands were identified using IFPRI’s Pilot Analysis of Global Ecosystem (PAGE) agricultural extent (PAGE v.1) [25].
5. Degrading or improving land was determined using University of Maryland’s Global Land Cover Facility’s AVHRR Global Production Efficiency Model (GloPEM) [24], which is available from 1981-2000 with annual summations of net primary production (NPP) change measured in grams of carbon sequestered per square meter per year (gC/$m^{2}$/yr). Consistent with previous studies [10,11], annual changes in climate-adjusted net primary productivity are taken as an indicator of land degradation or improvement.
6. Market accessibility was used to identify remote areas using a standard measure as released by the Global Environment Monitoring Unit of the Joint Research Centre of the European Commission [16]. Market access is identified as less than five hours of travel to a market city with a population of 50,000 or more. This dataset was published in seconds of travel to the nearest city and was converted to hours of travel. The resolution of this GRID formatted raster is 0.0083333333 by 0.0083333333 decimal degrees or 30 arc-seconds (approximately 1 km^2^ cells).

*Raster dataset management*

Our geospatial analysis was conducted using ESRI ArcGIS 10.1. All of the raster datasets used in this analysis were resampled to 30 arc-second ERDAS IMAGINE (.img) formatted raster layers using the nearest neighbor resampling technique.

*Maps and tables of spatial data*

All accompanying maps are projected using a standard Robinson (world) projection.

Figure A shows the global distribution per km^2^ of the rural population in developing countries in 2000 on all degrading agricultural land.

Figure B shows the global distribution per km^2^ of the rural population in developing countries in 2010 on all degrading agricultural land.

Figure C shows the global distribution per km^2^ of the rural population in developing countries in 2000 on all improving agricultural land.

Figure D depicts the global distribution per km^2^ of the rural population in developing countries in 2010 on all improving agricultural land.

Table A indicates our estimates of the rural population on all degrading agricultural lands in 2010.

Table B indicates our estimates of the rural population on all improving agricultural lands in 2010.

*Individual country data*

A complete data set for the 2000 and 2010 rural populations on DAL and IAL for each individual country can be found at the following web links:

<http://www.edwardbbarbier.com/Projects/ELD/Economics_of_Land_Degradation_data_2000DAL.html>

<http://www.edwardbbarbier.com/Projects/ELD/Economics_of_Land_Degradation_data_2010DAL.html>

<http://www.edwardbbarbier.com/Projects/ELD/Economics_of_Land_Degradation_data_2000_to_2010DAL.html>

*Statistical analysis of poverty*

Our statistical analysis examines whether the various spatial distributions of rural populations on degrading agricultural land (DAL) and improving agricultural land (IAL) in 2000 affect changes in the rate of poverty from 2000 to 2012 in 83 developing countries. Following a standard approach in the literature [18], we test whether this influence on poverty is direct, or whether it occurs through attenuating the poverty-reducing impact of income growth.

We consider four spatial distribution variables for the rural population in 2000 on degrading and improving agricultural land and remote areas for developing countries:

- the share (%) of rural population located on all degrading agricultural land (*d*_1_);
- the share (%) of rural population located on all remote degrading agricultural land (*d*_2_);
- the share (%) of rural population located on all improving agricultural land (*i*_1_); and
- the share (%) of rural population located on all remote improving agricultural (*i*_2_).

We obtain our cross-country measures of a given poverty line *z,* the poverty headcount index *H*, and mean income μ from PovcalNet, the on-line tool for poverty measurement developed by the Development Research Group of the World Bank (Available online at <http://iresearch.worldbank.org/PovcalNet/>). PovcalNet produces internationally comparable country-level poverty and income distribution estimates based on more than 850 standardized household surveys across 127 developing countries. From this database, we identify 83 low and middle-income economies with at least two suitable household surveys from 2000 to 2012. The longest available spell between surveys is used for each country, and both surveys use the same welfare indicator, either consumption or income per person. The median interval between surveys is eight years, and it varies from two to eleven years. As far as possible, the initial survey year chosen was 2000, or for the soonest subsequent year. However, for Burundi, Gambia, Ghana, Iran, Maldives and Yemen, the initial survey year was 1998, and for Kenya 1997. All monetary measures are in constant 2005 prices and are at Purchasing Power Parity (PPP).

The poverty headcount index *H* is the percentage of the population living in households with consumption per capita (or income when consumption is not available) below the poverty line. We follow a previous study [18] and choose a poverty line *z* of $2.00 per person per day at 2005 PPP, which is the median poverty line among developing countries. In the initial survey year, the median poverty headcount index across all 83 countries was 42.85%, but ranged widely from 0.29% to 95.44%. By the final survey year, the median poverty headcount was 27.86%, and it varied from 0.08% to 93.49%.

Mean income μ is the average monthly (2005 PPP $) per capita income or consumption expenditure from the household surveys for each country in the relevant year. In the initial survey year, the median per capita monthly income was $100 across all 83 countries, and ranged from $24 to $2,003. In the final survey year, median income was $115, and varied from $28 to $2,012. Finally, inequality is measured by the Gini index, which was also obtained from the PovcalNet cross-country household surveys for the relevant years.

Table C summarizes the descriptive statistics for the key variables used in the poverty analysis for our sample of 83 countries. This table is replicated as Table 3 in the main text.

We also employ a number of control variables in our analysis, following the approach of similar poverty analyses [18-21]. The controls are inflation, government consumption as a share of GDP, arable land per capita, agricultural value added as a share of GDP and per worker, investment as a share of GDP, trade openness, primary school enrollment, and life expectancy. These variables were obtained from the World Bank’s World Development Indicators (Available at <http://databank.worldbank.org/data>), and as far as possible, for 2000 and our sample of 83 countries. Other controls include a dummy for landlocked country as defined by UNDP (<http://unctad.org/en/pages/aldc/Landlocked%20Developing%20Countries/List-of-land-locked-developing-countries.aspx>), for small island developing states as defined by UNESCO (<http://www.unesco.org/new/en/natural-sciences/priority-areas/sids/about-unesco-and-sids/sids-list/>), and distance from equator for each country. We also employ rule of law and democracy (voice and accountability) indices, from the Worldwide Governance Indicators (<http://data.worldbank.org/data-catalog/worldwide-governance-indicators>), which were averaged over 1996-2000 for each country. Finally, we use regional dummies for the six main developing country regions, and the Gini index obtained from the PovcalNet surveys, as additional controls.

To analyze the possible direct and indirect influences of our spatial distribution variables *d_k_* and *i_k_* in 2000 on poverty changes from 2000 to 2012 in our 83 sample countries, we follow a similar estimation strategy of a previous study [18]. Thus, our basic regression is

$\gamma_{j}\left(H_{jt}\right)=\alpha_{0}+\alpha_{1}\ln\left(v_{jt-\tau}\right)\gamma_{j}\left(\mu_{jt}\right)+\left(\beta_{0}+\beta_{1}v_{jt-\tau}\right)\gamma_{j}\left(\mu_{jt}\right)+\omega_{jt}$, (1)

where *j* is each country observation, *t* is the final survey date, τ is the length of spell between surveys, and$\omega_{jt}$is the error term.

In equation (1), the dependent variable of the regression is, which is the annualized change in log headcount poverty rate for $2 a day between surveys, and thus represents growth in poverty. As noted above, across our sample of 83 countries, the median survey spell is eight years, and it varies from two to eleven years (i.e., the full 2000-2012 period).

A standard hypothesis in poverty analysis is that changes in poverty over time will be influenced by growth in income [18-21].. That is, as the mean per capita income across surveyed households rises, one would expect their average poverty rate to fall. Thus, a key explanatory variable in determining changes in poverty between surveys in equation (1) is the annual growth in income per person, which is represented by the annualize change in log survey mean income between surveys$\gamma_{j}\left(\mu_{jt}\right)\equiv\ln\left(\mu_{jt}/\mu_{jt-\tau}\right)/\tau$.

In this analysis, we are also interested in how a key spatial distribution variable of interest$v_{jt-\tau}$ also influences the change in poverty over time. As noted above, in estimating (1) we represent$v_{jt-\tau}$by one of our four spatial distribution variables in 2000, i.e.$d_{kj}, k=1,2$for degrading agricultural land and $i_{kj}, k=1,2$for improving agricultural land (see also Table S3). As indicated in equation (1), each of these spatial distribution variables could have a *direct impact* on changes in poverty over time$\alpha_{1}\ln\left(v_{jt-\tau}\right)\gamma_{j}\left(\mu_{jt}\right)$or it could affect the poverty-reducing impact of income growth$\left(\beta_{0}+\beta_{1}v_{jt-\tau}\right)\gamma_{j}\left(\mu_{jt}\right)$.

Two tests of restrictions on the various parameters estimated for equation (1) determine the direct and indirect influence of each$v_{jt-\tau}$on the annualized change in poverty. For example, rejection of the null hypothesis$\alpha_{1}=0$indicates that our spatial distribution variables$d_{k}$and$i_{k}$in 2000 have a direct influence on changes in poverty from 2000 to 2012, and subsequently, the magnitude of$\alpha_{1}$determines whether this influence is positive or negative. Failure to reject the null hypothesis of homogeneity, i.e.$\beta_{0}+\beta_{1}=0$in the case of any$d_{k}$and$\beta_{0}-\beta_{1}=0$in the case of$i_{k}$, confirms that initial spatial distributions of people on degrading or improving agricultural land have an indirect influence through affecting how income growth reduces poverty. If both of these restrictions hold, then the correct regressor in equation (1) is $\left(1-d_{kj}/100\right)\gamma\left(\mu_{jt}\right)$in the case of any DAL spatial distribution, and$\left(1+i_{kj}/100\right)\gamma_{j}\left(\mu_{jt}\right)$in the case of any IAL spatial distribution. Note also that, since the effect of income growth is to reduce poverty, the expected sign of the estimated parameter$\beta_{1}$is negative.

We conduct both OLS and IV regressions of (1), with the latter estimations using the growth rate in private consumption per capita obtained from the World Bank’s World Development Indicators (Available at <http://databank.worldbank.org/data>) as the instrument for the growth in mean survey income. This instrument has been used in previous poverty analysis studies [18, 21], as its use takes into account the possibility of a spurious negative correlation resulting from common measurement errors, given that the poverty measure and the mean per capita monthly income are based on the same household surveys.

In all OLS and IV regressions of (1), the null hypothesis$\alpha_{1}=0$cannot be rejected, which indicates that our spatial distribution variables$d_{k}$and$i_{k}$in 2000 do not have a direct influence on changes in poverty from 2000 to 2012. The homogeneity restrictions, i.e. $\beta_{0}+\beta_{1}=0$in the case of any$d_{k}$and$\beta_{0}-\beta_{1}=0$in the case of$i_{k}$, also cannot be rejected. This confirms that initial spatial distributions in 2000 of rural populations on DAL or IAL influence indirectly changes in poverty from 2000 to 2012 through affecting the impact of income growth on poverty reduction. The correct estimates of the poverty-reducing impact of growth are$\beta_{1}\left(1-d_{kj}/100\right)\gamma\left(\mu_{jt}\right)$for DAL and $\beta_{1}\left(1+i_{kj}/100\right)\gamma_{j}\left(\mu_{jt}\right)$for IAL, where the annualized growth rate in survey income per capita$\gamma\left(\mu\right)$ is evaluated at the mean for the sample of 83 countries (Table S3).

Tables D and E report the results for the estimated parameters$\alpha_{1}$ and $\beta_{1}$, and the relevant test statistics, for the OLS estimations of (1). These estimates are all significant and the correct sign, and did not change whether the control variables described above were included or excluded. In contrast, the IV elasticity estimates were generally not significant, and so are not indicated in the table.

In addition, a previous study [18] finds evidence that the initial variable of interest$v_{jt-\tau}$in equation (1) could be the initial poverty level$H_{jt-\tau}$. We replicate this analysis using the initial poverty level$H_{jt-\tau}$for $v_{jt-\tau}$in equation (1), for our sample of 83 countries over 2000 to 2012. As before, the null hypothesis $\alpha_{1}=0$and homogeneity restrictions cannot be rejected, which suggest that the initial level of poverty$H_{jt-\tau}$has no direct influence on poverty changes over time and the poverty-adjusted growth rate$\left(1-H_{jt-\tau}/100\right)\gamma\left(\mu_{jt}\right)$ is the relevant regressor in equation (1). Our replication of the estimations in [18] for our sample of 83 countries produces similar results. When homogeneity is imposed on (1), we obtain a significant and negative poverty-adjusted growth elasticity, which is -2.83 for OLS and -4.85 for IV. The corresponding estimates in [18] are -2.47 and -3.09, respectively.

Finally, allowing for the possibility that the initial level of poverty$H_{jt-\tau}$is endogenous with respect to our four spatial distribution measures, we estimate instrumental variable (IV), seemingly unrelated regressions (SUR) and three-stage least squares (3SLS) regressions of equation (1) taking this endogeneity into account. Thus, for these regressions, the relevant system of equations is

$\gamma_{j}\left(H_{jt}\right)=\alpha_{0}+\beta_{1}\left(1-H_{jt-\tau}/100\right)\gamma_{j}\left(\mu_{jt}\right)+\omega_{jt}$, (2)

$\left(1-H_{jt-\tau}/100\right)\gamma_{j}\left(\mu_{jt}\right)=\delta_{0}+\delta_{1}\left(1-d_{kj}/100\right)\gamma_{j}\left(\mu_{jt}\right)+\varepsilon_{jt}, k=1,2$ (3a)

$\left(1-H_{jt-\tau}/100\right)\gamma_{j}\left(\mu_{jt}\right)=\delta_{0}+\delta_{1}\left(1+i_{kj}/100\right)\gamma_{j}\left(\mu_{jt}\right)+\varepsilon_{jt}, k=1,2$. (3b)

As before, the expected sign of $\beta_{1}$is negative, whereas the expected sign of $\delta_{1}$is positive. The latter result implies that in (3a)$d_{k}$is positively correlated with$H_{jt-\tau}$, and in (3b)$i_{k}$is negatively correlated with$H_{jt-\tau}$. It follows from (2) and (3a) that the correct estimate of the poverty-reducing impact of growth for DAL is$\beta_{1}\delta_{1}\left(1-d_{kj}/100\right)\gamma\left(\mu_{jt}\right)$, and correspondingly from (2) and (3b), the correct estimate for IAL is$\beta_{1}\delta_{1}\left(1+i_{kj}/100\right)\gamma_{j}\left(\mu_{jt}\right)$. The annualized growth rate in survey income per capita$\gamma\left(\mu\right)$ is again evaluated at the mean for the sample of 83 countries.

Tables D and E report the parameter estimates for $\beta_{1}$and $\delta_{1}$and relevant test statistics for the 3SLS regressions, although the results are also robust for SUR and IV, the coefficients significant, and with the expected signs. The results are also robust when additional control variables are included in the regressions, although almost all the controls are individually and jointly insignificant in all IV, SUR and 3SLS regressions, with the exception of agricultural value added per worker, investment as a share of GDP and the Europe and Central Asia dummy. Income inequality as measured by the Gini index is always insignificant when included as a separate control variable.

Table 4 in the main text combines the 3SLS parameter estimates for $\beta_{1}$and $\delta_{1}$, with and without controls, from Tables D and E with the mean and standard deviation estimates for each spatial distribution variable as reported in Table S3 to derive different estimates of the effects of share of rural population on DAL on the poverty-reducing impact of growth $\beta_{1}\delta_{1}\left(1-d_{kj}/100\right)\gamma\left(\mu_{jt}\right)$ and the corresponding effects for the share of rural population on IAL $\beta_{1}\delta_{1}\left(1+i_{kj}/100\right)\gamma_{j}\left(\mu_{jt}\right)$.


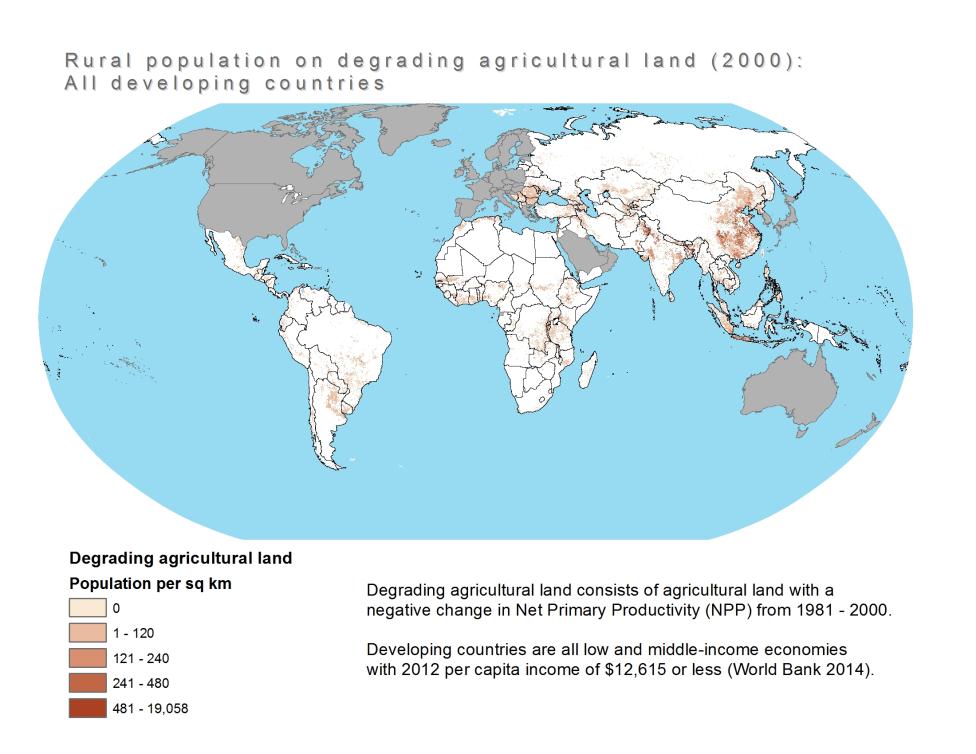


**Figure A. Distribution of rural population of developing countries on all degrading agricultural land, 2000**

Developing countries are all low and middle-income economies with 2012 per capita income of US$12,615 or less, as defined by the World Bank’s World Development Indicators (Available at <http://databank.worldbank.org/data>).

**
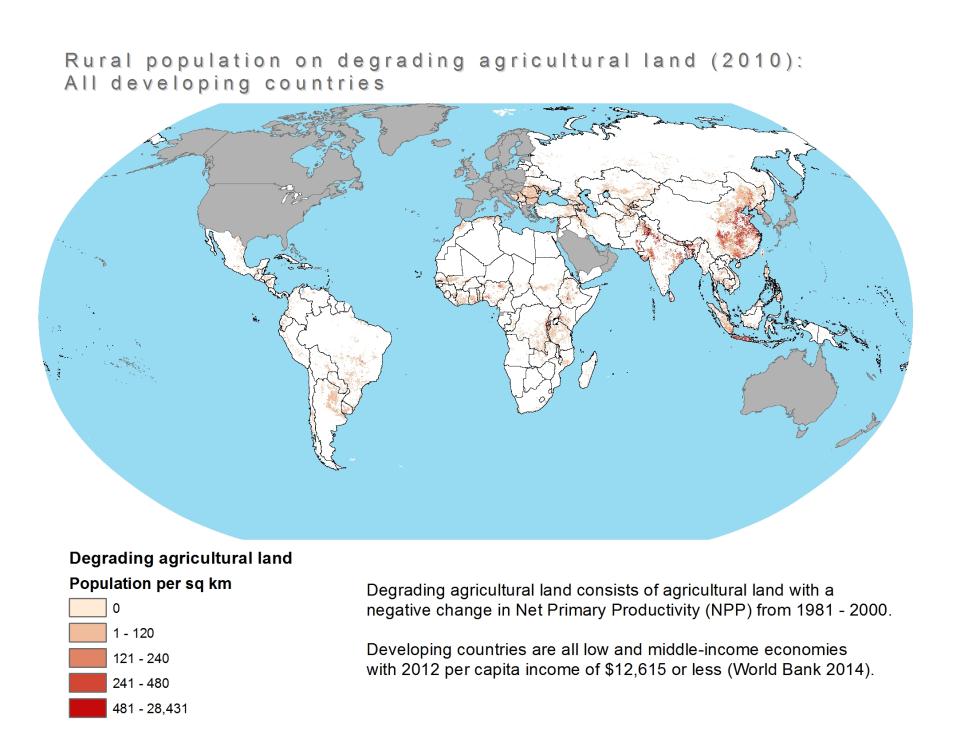
**

**Figure B. Distribution of rural population of developing countries on all degrading agricultural land, 2010**

Developing countries are all low and middle-income economies with 2012 per capita income of US$12,615 or less, as defined by the World Bank’s World Development Indicators (Available at <http://databank.worldbank.org/data>).

**
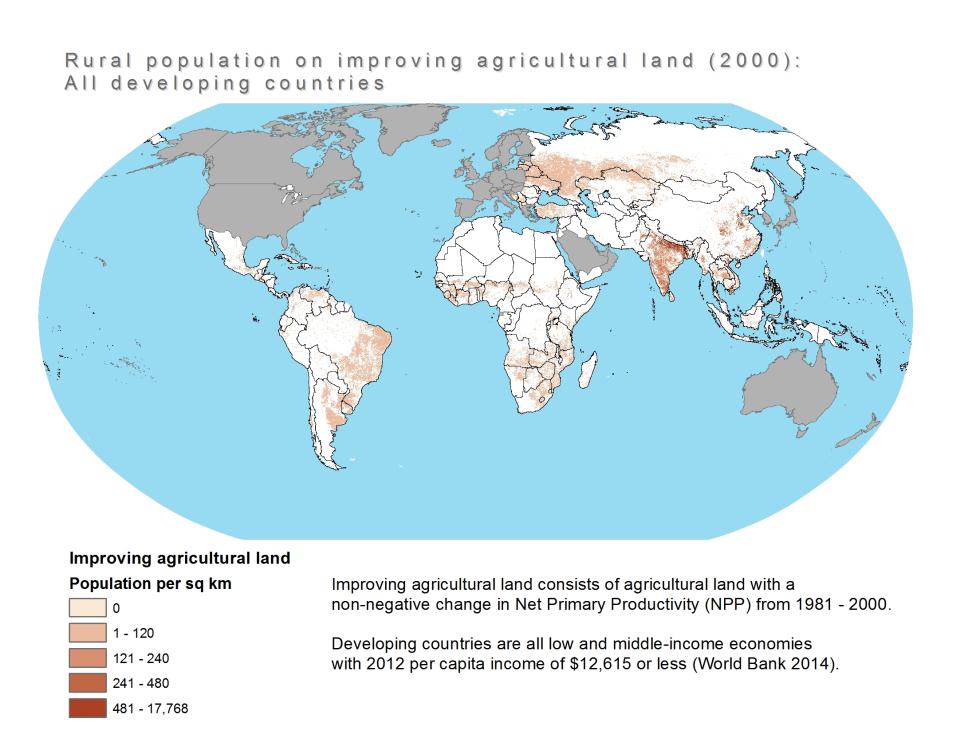
**

**Figure C. Distribution of rural population of developing countries on all improving agricultural land, 2000**

Developing countries are all low and middle-income economies with 2012 per capita income of US$12,615 or less, as defined by the World Bank’s World Development Indicators (Available at <http://databank.worldbank.org/data>).
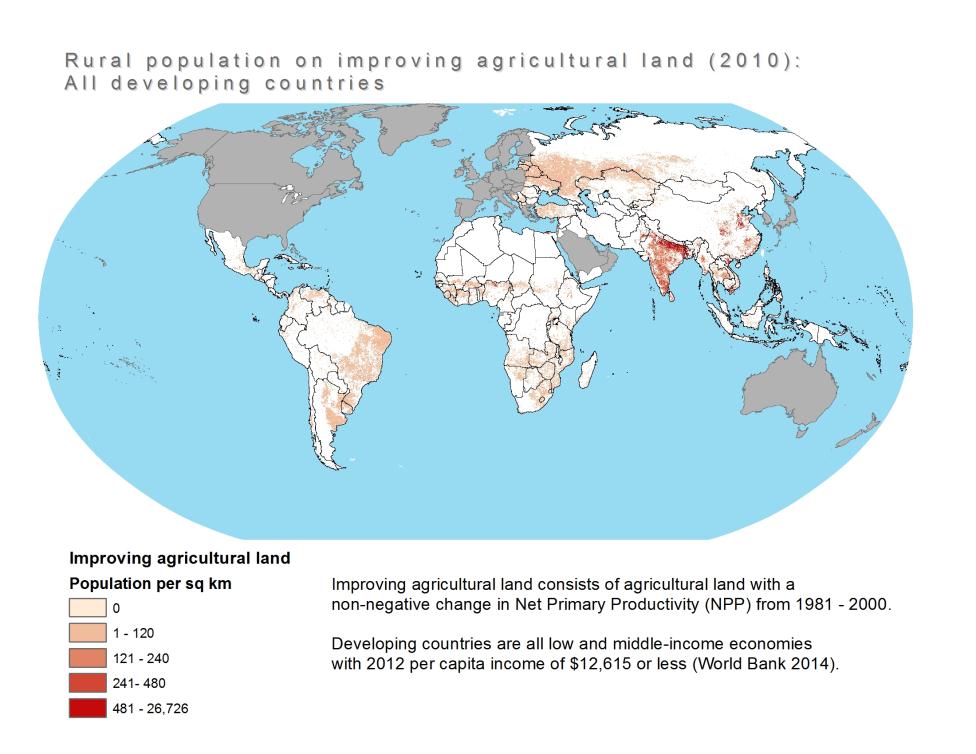


**Figure D. Distribution of rural population of developing countries on all improving agricultural land, 2010**

Developing countries are all low and middle-income economies with 2012 per capita income of US$12,615 or less, as defined by the World Bank’s World Development Indicators (Available at <http://databank.worldbank.org/data>).**Table A. Rural population on all degrading agricultural lands, 2010**

|  | **Population in 2010 (millions)** | | | | |
| --- | --- | --- | --- | --- | --- |
|  | Rural population  (1) | Rural population  on all DAL  (2) | % share  (2)/(1) | Rural population  on all remote DAL  (3) | % share  (3)/(1) |
| **Developing country**  East Asia & Pacific  Europe & C. Asia  Latin America & Caribbean  Middle East & N. Africa  South Asia  Sub-Saharan Africa | **4,248.6**  1,499.1  180.7  336.1  237.2  1,284.0  711.4 | **1,426.3**  770.1  67.7  45.3  49.9  336.1  157.2 | **33.6 %**  51.4 %  37.4 %  13.5 %  21.1 %  26.2 %  22.1 % | **230.2**  133.6  6.5  6.6  5.8  32.6  45.1 | **5.4 %**  8.9 %  3.6 %  2.0 %  2.4 %  2.5 %  5.4 % |
| **Developed country** | **415.3** | **70.6** | **17.0 %** | **3.1** | **0.7 %** |
| **World** | **4,663.9** | **1,496.9** | **32.1 %** | **233.2** | **5.0 %** |

Degrading agricultural land (DAL) consists of agricultural land with a negative change in Net Primary Productivity (NPP) from 1981-2000. NPP is measured as the change in grams of carbon sequestered per m^2^ over the 1981-2000 time period after subtracting respiration losses. Market accessibility is used to identify remote DAL, where market access is defined as less than five hours of travel to a market city with a population of 50,000 or more.

Developing countries are all low and middle-income economies with 2012 per capita income of US$12,615 or less, as defined by the World Bank’s World Development Indicators (Available at <http://databank.worldbank.org/data>).

Column (1) is estimated for 205 countries. Columns (2) and (3) are estimated for 183 countries; one country was indeterminate due to changing political boundaries, and 21 countries had missing data or insufficient spatial resolution denoting agricultural land.

**Table B. Rural population on all improving agricultural lands, 2010**

|  | **Population in 2010 (millions)** | | | | |
| --- | --- | --- | --- | --- | --- |
|  | Rural population  (1) | Rural population  on all IAL  (2) | % share  (2)/(1) | Rural population  on all remote IAL  (3) | % share  (3)/(1) |
| **Developing country**  East Asia & Pacific  Europe & C. Asia  Latin America & Caribbean  Middle East & N. Africa  South Asia  Sub-Saharan Africa | **4,248.6**  1,499.1  180.7  336.1  237.2  1,284.0  711.4 | **1,539.4**  446.3  66.3  103.3  34.6  734.5  154.3 | **36.2%**  2.8%  36.7%  30.7%  14.6%  57.2%  21.7% | **169.2**  68.2  7.0  10.5  2.5  43.8  37.2 | **4.0%**  4.5%  3.9%  3.1%  1.1%  3.4%  5.2% |
| **Developed country** | **415.3** | **190.5** | **45.9%** | **9.0** | **2.2%** |
| **World** | **4,663.9** | **1,729.9** | **37.1%** | **178.2** | **3.8%** |

Improving agricultural land (IAL) consists of agricultural land with a non-negative change in Net Primary Productivity (NPP) from 1981-2000. NPP is measured as the change in grams of carbon sequestered per m^2^ over the 1981-2000 time period after subtracting respiration losses. Market accessibility is used to identify remote IAL, where market access is defined as less than five hours of travel to a market city with a population of 50,000 or more.

Developing countries are all low and middle-income economies with 2012 per capita income of US$12,615 or less, as defined by the World Bank’s World Development Indicators (Available at <http://databank.worldbank.org/data>).

Column (1) is estimated for 205 countries. Columns (2) and (3) are estimated for 183 countries; one country was indeterminate due to changing political boundaries, and 21 countries had missing data or insufficient spatial resolution denoting agricultural land.

**Table C. Descriptive statistics of key poverty analysis variables**

|  | **Descriptive Statistics** | | |
| --- | --- | --- | --- |
| Key variables | Mean | Median | Standard  Deviation |
| Initial headcount poverty rate ( % of population) in 2000, *H* | 46.41 | 42.85 | 29.56 |
| Annualized growth ( %) in the poverty rate (US$2/day) from 2000-2012, γ(*H*) | -7.70 | -4.26 | 10.28 |
| Annualized growth ( %) in the mean survey income per capita from 2000-2012, γ(μ) | 3.36 | 3.32 | 3.52 |
| % of rural population on all degrading agricultural land in 2000, *d*_1_ | 27.11 | 22.44 | 21.04 |
| % of rural population on all remote degrading agricultural land in 2000, *d*_2_ | 5.02 | 3.81 | 4.43 |
| % of rural population on all improving agricultural land in 2000, *i*_1_ | 31.89 | 29.6 | 21.05 |
| % of rural population on all remote improving agricultural land in 2000, *i*_2_ | 13.45 | 5.21 | 18.83 |

Based on a sample of 83 developing countries.**Table D. Key estimated parameters and statistical tests for *d_k_* variables**

1. **Share (%) of rural population on all degrading agricultural land in 2000, *d*_1_**

|  | **OLS** | | **3SLS** | | | |
| --- | --- | --- | --- | --- | --- | --- |
|  | α_1_ | β_1_ | β_1_ | | δ_1_ | |
|  |  |  | With  controls | Without  controls | With  controls | Without  controls |
| Parameter estimates | 0.008  (0.626) | -1.468  (-2.849)** | -2.147  (-3.830)** | -2.512  (-4.386)** | 0.540  (8.909)** | 0.584  (7.053)** |
| Observations (*N*) | 80 | 80 | 80 | 80 | 80 | 80 |
| *R*^2^ | 0.31 | 0.13 | 0.57 | 0.46 | 0.69 | 0.38 |
| Likelihood ratio test | 29.86** |  | 74.54** | 52.02** | 96.60** | 40.71** |
| *F*-test | 11.46** | 11.52** | 19.96** | 67.70** | 55.17** | 48.51** |
| Homogeneity test | 0.20 |  |  |  |  |  |

**B. Share (%) of rural population on all remote degrading agricultural land in 2000, *d*_2_**

|  | **OLS** | | **3SLS** | | | |
| --- | --- | --- | --- | --- | --- | --- |
|  | α_1_ | β_1_ | β_1_ | | δ_1_ | |
|  |  |  | With  controls | Without  controls | With  controls | Without  controls |
| Parameter estimates | 0.010  (1.371) | -1.664  (-4.731)** | -2.307  (-4.721)** | -2.911  (-6.417)** | 0.523  (12.62)** | 0.572  (10.895)** |
| Observations (*N*) | 80 | 80 | 80 | 80 | 80 | 80 |
| *R*^2^ | 0.30 | 0.27 | 0.57 | 0.48 | 0.79 | 0.60 |
| Likelihood ratio test | 28.11** |  | 74.45** | 53.83** | 129.11** | 74.81** |
| *F-*test | 10.67** | 28.76** | 19.92** | 71.05** | 95.54** | 115.73** |
| Homogeneity test | 0.10 |  |  |  |  |  |

OLS is ordinary least squares; 3SLS is three-stage least squares. *t*-ratios are in parentheses. **significant at the 1% level. The homogeneity test is the *t*-test for the restriction$\beta_{0}+\beta_{1}=0$in equation (1). Three countries had missing data.**Table E. Key estimated parameters and statistical tests for *i_k_* variables**

1. **Share (%) of rural population on all improving agricultural land in 2000, *i*_1_**

|  | **OLS** | | **3SLS** | | | |
| --- | --- | --- | --- | --- | --- | --- |
|  | α_1_ | β_1_ | β_1_ | | δ_1_ | |
|  |  |  | With  controls | Without  controls | With  controls | Without  controls |
| Parameter estimates | 0.012  (0.916) | -1.664  (-4.731) | -2.356  (-4.660)** | -2.916  (-6.113)** | 0.341  (11.551)** | 0.370  (9.695)** |
| Observations (*N*) | 80 | 80 | 80 | 80 | 80 | 80 |
| *R*^2^ | 0.27 | 0.27 | 0.57 | 0.48 | 0.77 | 0.54 |
| Likelihood ratio test | 25.06** |  | 74.36** | 53.84** | 119.97** | 64.19** |
| *F*-test | 9.32** | 28.76** | 19.88** | 71.06** | 82.49** | 91.65** |
| Homogeneity test | 0.39 |  |  |  |  |  |

**B. Share (%) of rural population on all remote improving agricultural land in 2000, *i*_2_**

|  | **OLS** | | **3SLS** | | | |
| --- | --- | --- | --- | --- | --- | --- |
|  | α_1_ | β_1_ | β_1_ | | δ_1_ | |
|  |  |  | With  controls | Without  controls | With  controls | Without  controls |
| Parameter estimates | 0.007  (0.868) | -1.080  (-3.792)** | -2.301  (-4.337)** | -2.859  (-5.604)** | 0.379  (10.114)** | 0.411  (8.470)** |
| Observations (*N*) | 80 | 80 | 80 | 80 | 80 | 80 |
| *R*^2^ | 0.32 | 0.24 | 0.57 | 0.48 | 0.72 | 0.47 |
| Likelihood ratio test | 30.60** |  | 74.46** | 53.74** | 107.36** | 53.24** |
| *F-*test | 11.80** | 25.23** | 19.92** | 70.88** | 66.76** | 69.95** |
| Homogeneity test | 6.74 |  |  |  |  |  |

OLS is ordinary least squares; 3SLS is three-stage least squares. *t*-ratios are in parentheses. **significant at the 1% level.

The homogeneity test is the *t*-test for the restriction$\beta_{0}-\beta_{1}=0$in equation (1). Three countries had missing data.
